# Mental Health Beliefs, Access, and Engagement with Military Sexual Trauma–Related Mental Health Care

**DOI:** 10.1007/s11606-022-07590-6

**Published:** 2022-08-30

**Authors:** Caitlin L. McLean, Jessica A. Turchik, Rachel Kimerling

**Affiliations:** 1 VA San Diego Healthcare System, San Diego, CA USA; 2 Department of Psychiatry, University of California San Diego, La Jolla, CA USA; 3 National Center for PTSD, Dissemination and Training Division, & Center for Innovation to Implementation, VA Palo Alto Health Care System, Menlo Park, CA USA

**Keywords:** military sexual trauma, barriers, access, engagement

## Abstract

**Background:**

Military sexual trauma (MST) is associated with negative mental health outcomes. Mental health beliefs, such as stigma or secondary victimization, have been identified as possible barriers to care; however, it is unclear whether such beliefs impact receiving care.

**Objective:**

To assess if mental health beliefs impact MST-related mental health care access and engagement.

**Design:**

Veterans completed a survey following detection of MST. Survey data were linked to Veteran's Health Administration administrative data in order to examine associations with outpatient MST-related care in the year following MST detection.

**Participants:**

A national sample of women and men Veterans (*N* = 1,185) with newly detected MST who reported a perceived need for MST-related mental health treatment.

**Main Measures:**

Building on prior work that identified four latent classes of mental health beliefs (Hahn et al., *J Trauma Stress* 34:394–404, 2020; *low barrier*, *stigma-related beliefs*, *negative perceptions of care*, *high barrier*), we examined associations with outpatient mental health care indicated by a provider as related to MST. Care was examined in total, as well as the specific categories of medication management, individual psychotherapy, and group psychotherapy.

**Key Results:**

Access to care following MST detection was high: most (71.6%) Veterans had an MST-related mental health visit within 30 days, and nearly all (83.6%) within 180 days, with the median number of days to receiving care being 2. *Negative perceptions of care* predicted a lower likelihood of treatment engagement (3+ encounters) with MST-related individual psychotherapy (*OR* = 0.65, 95% CI (0.43–0.96)), whereas the *stigma-related beliefs* and *high barrier* classes did not.

**Conclusions:**

There appears to be excellent access to mental health care among Veterans with a perceived need for MST-related mental health treatment. However, treatment beliefs that represented negative perceptions of care may serve as barriers to engagement. Interventions targeting negative perceptions of care during early encounters could help promote subsequent engagement.

## INTRODUCTION

Military sexual trauma (MST), or sexual assault and/or harassment that occurred while in the military, affects significant portions of both women and men Veterans. Approximately 41.5% of women and 4.0% of men who served during Operations Enduring Freedom and Iraqi Freedom (OEF/OIF) report MST.^1^ Surveillance data from the Veteran’s Health Administration (VHA) of users in 2020 indicate that 32.4% of women (141,365) and 1.9% of men (77,309) report a history of MST.^2^ MST experiences are associated with increased risks for chronic conditions and mental health conditions, with posttraumatic stress disorder (PTSD) and depression among the most frequent mental health consequences of MST.^3,4^ To promote access to care for MST-related conditions, the VHA has implemented universal screening for MST and provides care for related conditions without co-pay. VHA surveillance data for fiscal year 2020 indicate that 63.4% of women and 53.6% of men with newly detected MST accessed mental health care related to the MST.^2^ After assuring access, promoting engagement with MST-related mental health care is the next critical step in effective MST-related care.

Sexual trauma is an extremely private and stigmatizing experience contextualized by gender-linked societal schemas, often called “rape myths,” that normalize the experience perpetuating inaccurate beliefs that place responsibility on the victim (question the veracity of reports, minimize experiences, justify the perpetrator’s behavior).^5^ Survivors of MST frequently report feeling shame, embarrassment, and expectations of negative reactions from others. The latter is often called “secondary victimization” in reference to institutional personnel, such as military leadership, law enforcement, or health care providers, as endorsement of negative social schema by such individuals may be especially toxic. As a result, fears regarding being blamed or seen as responsible or not believed are particularly salient to treatment-seeking MST survivors.^6–11^

In a recent study of women and men Veterans with recently detected MST, Hahn et al.^12^ examined a wide range of such mental health beliefs related to MST and found 4 classes of beliefs. The same classes were constructed separately by gender using multiple group latent class analysis (LCA), so these classes represent gender-tailored treatment beliefs: *low barrier*, with few negative beliefs; *high barrier*, with extensive negative beliefs; *stigma-related beliefs*, including shame and embarrassment and others finding out; and *negative perceptions of care*, characterized by concerns about secondary victimization. Secondary victimization can also occur in the context of institutional betrayal, or harm caused by an institution to individuals dependent on that institution. Gender differences emerged in the composition of classes consistent with prior work. For women,^8,12,13^ the *stigma-related beliefs* class indicated heightened concerns regarding opposite-sex providers and *negative perceptions of care* indicated discomfort with the majority male environment of VHA settings. Among men,^6,12^ the *stigma-related beliefs* class revealed concerns about same-sex providers, as well as concerns regarding sexual orientation, and the *negative perceptions of care* class indicated heightened concerns about not being believed, perhaps associated with perceptions that sexual trauma occurs primarily among women Veterans.

The extent to which some or all of these beliefs about mental health and treatment pose a barrier to care is unclear. To date, varied populations and study designs demonstrate disparate findings. Stigma was associated with lower treatment utilization in the prior 6 months in a retrospective study of active-duty soldiers with a history of sexual assault.^14^ Yet, in a similar cross-sectional study of VHA users with probable PTSD and/or depression, stigma was not associated with seeking care, though negative beliefs about treatment-seeking were associated with a lower likelihood of use.^15^ In a retrospective study of women VHA users with a history of MST, a study found that those who reported *more* institutional betrayal were *more* likely to have used VHA care in the year prior.^16^ Notably, the aforementioned studies all evaluated care receipt that occurred prior to surveying beliefs. It may be that people who receive enough care to benefit also report more positive beliefs. One prospective evaluation following PTSD diagnosis found that neither stigma nor negative perceptions of care were associated with initiating psychotherapy.^17^ Surprisingly, those with greater stigma were more likely to engage with care and completed more visits. ^17^

The current study aims to clarify relationships between mental health beliefs and access to and engagement with care by using a quasi-prospective design to examine beliefs about MST-related mental health after detection of MST via screening, and examining receipt of MST-related mental health care over the year following screening. Understanding the extent to which negative treatment beliefs, or specific types of treatment beliefs, may present a barrier to MST-related care is crucial to shaping trauma-informed care for MST survivors. Moreover, VHA’s proactive policies such as the co-pay release for MST-related care create an ideal context to explore the role of treatment beliefs in engagement due to the lack of barriers to disclosure or limited availability of mental health services that are common to other health care systems. We extend Hahn et al.’s^12^ analysis of mental health beliefs to determine the impact on access to and engagement with MST-related care. We conceptualize the study within Andersen’s Behavioral Model of Health Services Use,^18^ which asserts that use of health care services is a function of predisposing factors (preexisting and belief characteristics), need factors (symptom severity), and enabling factors (those that support or impede use). The primary aim of this study was to examine mental health belief classes as predictors of access to and engagement with MST-related mental health care (medication management visits, individual psychotherapy, and group psychotherapy) among a national sample of women and men Veterans with newly detected MST and who reported a perceived need for care.

## METHODS

### Design and Procedures

This study is a re-analysis of the Understanding Veterans’ Opinions and Attitudes about VA Health Care Survey, a sample of 2,220 women and men Veterans with recently detected MST. We use a quasi-prospective design, recruiting Veterans for a mail survey within 3 months of newly detected MST, and following treatment utilization for the year following screening. Veterans were randomly sampled from facilities in the continental U.S. between August 2013 and March 2014. The VHA MST screen in the electronic medical record includes the following: “While you were in the military: 1) Did you ever receive uninvited and unwanted sexual attention, such as touching, cornering, pressure for sexual favors, or verbal remarks?; 2) Did someone ever use force or threat of force to have sexual contact with you against your will?” An affirmative response to either question results in a positive screen. The current study is an extension of Hahn et al.,^12^ a cross-sectional study that identified latent classes of mental health beliefs among a subsample of 1,185 Veterans who endorsed perceived need for MST-related mental health care by an affirmative response to the survey item: “In the past year, did you ever want or need help with any emotional or mental health concerns related to any military sexual trauma experience?” We linked those survey data with VHA administrative data in order to examine associations with outpatient MST-related care in the year following MST detection.

### Participants

The sample consisted of 796 women and 389 men (*N* = 1,185). Demographic characteristics for the sample are presented in Table 1.

**Table 1:** Demographics, Barrier Class, and Relevant Variables by Gender

Characteristic	All		Women		Men	
	*n*	%	*n*	%	*n*	%
	1185	100	796	67.2	389	32.8
Age						
18-44	402	33.9	346	44.1	56	14.8
45-64	678	57.2	420	53.5	258	68.1
65+	84	7.1	19	2.4	65	17.2
Race						
White	721	60.8	471	59.7	250	65.1
Black/African American	326	27.5	236	29.9	90	23.4
Asian/Asian American	15	1.3	11	1.4	4	1.0
American Indian/Alaska Native	13	1.1	9	1.1	4	1.0
Native Hawaiian/Pacific Islander	5	0.4	3	0.4	2	0.5
Other/mixed race	93	7.9	59	7.5	34	8.9
Ethnicity						
Latinx	98	8.3	66	8.3	32	8.2
Sexual orientation						
Heterosexual	1024	88.2	695	88.9	329	86.8
Gay/lesbian/bisexual	121	10.4	78	10.1	43	11.3
Other	16	1.4	9	1.2	7	1.8
OEF/OIF^a^	265	22.4	224	28.3	41	10.6
Mental Health Beliefs						
Low barrier class	365	30.8	281	35.3	84	21.6
Stigma-related beliefs class	402	33.9	271	34.0	131	33.7
Negative perceptions of care class	95	8.0	57	7.2	38	9.8
High barrier class	323	27.3	187	23.5	136	35.0
Past-year mental health care use	598	50.5	346	43.5	252	64.8
Positive mental health screen	1050	88.6	692	86.9	358	92.0
One logistical barrier	408	34.4	277	34.8	131	33.7
Two+ logistical barriers	287	24.2	213	26.8	74	19.0

*Note.* Percentages are valid percent

^a^Served during Operation Enduring Freedom and Operation Iraqi Freedom

### Data Sources

#### Survey

The Understanding Veterans’ Opinions and Attitudes about VA Health Care Survey was designed to assess perceived need, perceived barriers, and access to and satisfaction with MST-related mental health care. The survey assessed patient characteristics (e.g., demographics, mental health screens), and mental health services preferences.

##### Mental Health Beliefs

The survey included 20 items assessing MST-related mental health beliefs, such as “I would feel ashamed to talk to my provider about MST” and “Tough people can handle their problems on their own.” Instructions for these items were specifically focused on MST-related care. We examined the latent classes of mental health beliefs as constructed by Hahn et al.^12^ Using a multiple-group (with gender as the grouping variable) LCA, Hahn et al.^12^ identified four mutually exclusive groups of treatment belief classes: *low barrier* class (32.4%); *stigma-related beliefs* class (34.1%); *negative perceptions of care* class (10.1%); and *high barrier* class (23.5%). Model fit indices indicated that the four-class solution was the superior fit; sample size adjusted Bayesian information criteria = 5,219.66 and entropy = .78.

##### Clinically Significant Symptoms

We used two screening questionnaires to assess for the presence of PTSD and depression symptoms. The Primary Care PTSD Screen^19^ assessed probable PTSD. Participants screened positive if they endorsed experiencing a potentially traumatic event and a minimum of three of four items measuring hyperarousal, avoidance, re-experiencing, and numbing in the past month. A cutoff score of 3 is considered optimally efficient in a Veteran sample (sensitivity = 78.0; specificity = 87.0).^19^ The current data had excellent internal consistency (*α* = .96). Depressive symptoms were assessed using the Patient Health Questionaire-2.^20^ Participants screened positive if they scored 2 or higher on two items rated from 0 (*not at all*) to 3 (*nearly every day*) that assessed depression symptoms over the past 2 weeks. A cutoff score of 2 has adequate sensitivity (82.1) and specificity (80.4) for detecting depressive disorders.^20^ The current data had good internal consistency (*α* = .84). We created a variable that indicated if a respondent met criteria for one or more screens.

##### Logistical Barriers

Four items assessed perceived logistical barriers (e.g., childcare, work, transportation, scheduling difficulties). We created a variable that indicated endorsement of no barriers, one barrier, and two or more barriers.

#### VHA Administrative Data

National Patient Care Database outpatient treatment files were used to characterize utilization in the year following MST detection. For Veterans with a positive screen for MST, the VHA electronic medical record prompts providers to denote whether the care provided during the visit is related to the MST experiences when entering diagnostic and procedure codes.^21^ This indicator identifies encounters for co-pay release but can also be used to track the clinical care provided for MST-related conditions, or MST-related care. We aggregated outpatient mental health encounters for all MST-related care. We also examined three subsets of these encounters, using definitions consistent with VHA performance measurement,^22^ hierarchically coded as follows: individual psychotherapy (cpt codes: 90801, 90806, 90818, 90808, 90821, 90807, 90819, 90822, 90845, 90791, 90834, 90837); group psychotherapy (cpt codes: 90853, 90857); and medication management (mental health visit where the provider has a prescriber person-class that is not coded as psychotherapy). Because the sample is comprised of Veterans with a perceived need for care, we considered one or more visits to indicate access to care, and three or more visits to indicate engagement with care, consistent with prior work.^23^

### Data Analysis

#### Access

We first examined access to MST-related mental health visits in the year after MST detection. This included the percentage of individuals who had one or more visits, describing number of days to access and number of visits, in the year after detection. Because access was universally high, we did not model access as a function of latent classes of treatment beliefs.

#### Engagement with MST-Related Mental Health Care

We modeled engagement with MST-related mental health care as a function of latent class membership. Our goal was to model these relationships while minimizing potential bias due to the following: classification uncertainty in the LCA,^24^ confounding due to group differences across classes,^25^ and effects of clustering within VHA facilities. The latent classes of treatment beliefs demonstrated reliable class separation (mean class posterior probabilities > .80), suggesting bias due to class assignment is unlikely.^24^ We adjusted standard errors to address clustering within facilities and used theoretically informed hierarchical regressions to demonstrate the effects of baseline class differences that could serve as predisposing or enabling factors to MST-related mental health treatment engagement. Blocks of variables were sequentially entered to illustrate effects for the following: gender; predisposing factors (treatment beliefs, mental health use in the year prior to MST detection); need (clinically significant symptoms of PTSD or depression); and enabling factors (single or multiple logistical barriers to care). We conducted post hoc sensitivity analyses to test for potential bias in results from the small subset of Veterans who completed all MST-related care within the 3 months following screening, for whom the report of treatment beliefs was less likely to have preceded engagement with care.

## Results

### Access

Of the 1,185 Veterans with perceived need for MST-related mental health care, 1,052 (88.8%) accessed MST-related mental health care in the year after MST detection, with access similarly high among both women and men. Of these individuals, 85.0% (*n* = 894) attended at least one medication management visit, 81.8% (*n* = 861) attended individual psychotherapy, and 36.0% (*n* = 379) group psychotherapy. Most Veterans participated in more than one treatment modality: 25.3% (*n* = 266) used only one form of care, 44.1% (*n* = 464) used two of these forms of care, and 30.6% (*n* = 322) used all three. The median number of MST-related mental health visits attended was 12 (IQR = 5.00–25.75) and the modal number of visits was 4.

**Figure 1:**
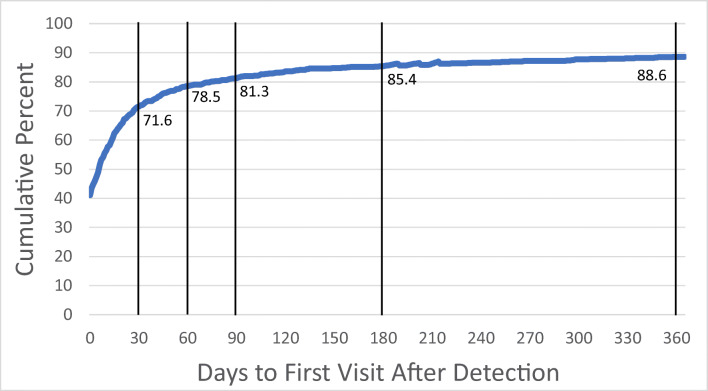
**Days to access first MST-related mental health care visit (*N*** **= 1,185)**

Most individuals (71.9%) received MST-related mental health care within the first 30 days after screening. The median number of days to receiving care was 2 days with an interquartile range (IQR) of 0.00 to 21.00 days. The modal interval was 0, indicating most initial encounters represented same-day access. Figure 1 illustrates the timeliness of access to MST-related care.

### Engagement

Models predicting engagement with MST-related mental health care are shown in Table 2. Gender differences emerged only for the unadjusted model, where women were less likely to engage compared to men. When predisposing and need factors were examined, the *negative perceptions of care* class emerged as a barrier to engagement, with about half the odds of engaging with care compared to the low barrier class. In the enabling factors step, *negative perceptions of care* failed to achieve statistical significance in the presence of logistical barriers, which demonstrated strong negative effects on engagement. Clinically significant symptoms of depression or PTSD increased the likelihood of engagement, as did past-year mental health care. Logistical barriers predicted a lower likelihood of engagement; however, *negative perceptions of care* class dropped out as a significant predictor, suggesting that the presence of logistical barriers may be more proximal predictors to decreasing engagement than mental health beliefs. We repeated analyses excluding 96 Veterans who completed all MST-related mental health care within the first 90 days following screening and results did not significantly differ.

**Table 2:** Hierarchical Regressions Predicting Engagement with MST-Related Mental Health Care (*n*=1,048)

	Gender AOR (95% CI)	Predisposing Factors AOR (95% CI)	Need AOR (95% CI)	Enabling Factors AOR (95% CI)
Pseudo *R*^2^	.005	.081	.098	.118
Gender				
Men	ref	ref	ref	ref
Women	**0.69 (0.49–0.96)**	0.95 (0.66–1.39)	0.99 (0.67–1.45)	1.07 (0.73–1.57)
Mental health beliefs				
Low barrier class		ref	ref	ref
Stigma-related beliefs class		1.04 (0.72–1.52)	0.98 (0.67–1.43)	1.15 (0.78–1.70)
Negative perceptions of care class		**0.59 (0.36–0.98)**	**0.52 (0.31–0.87)**	0.71 (0.40–1.26)
High barrier class		0.81 (0.55–1.20)	0.69 (0.46–1.02)	0.92 (0.59–1.43)
Past-year mental health care use				
No		ref	ref	ref
Yes		**4.90 (1.76–4.11)**	**4.90 (3.37–7.12)**	**2.90 (1.84–4.57)**
Clinically significant symptoms				
No positive screen			ref	ref
Positive mental health screen			**2.67 (1.71–4.17)**	**4.65 (3.17–6.82)**
Logistical barriers to care				
None				ref
One logistical barrier				**0.53 (0.35–0.80)**
Two+ logistical barriers				**0.37 (0.24–0.57)**

*Note.* Adjusted for 128 stations. *AOR* adjusted odds ratio, *ref* reference category

Significant AORs are presented in bold

Results for medication management visits are displayed in Table 3. Gender was the only significant predictor, with women less likely than men to engage. Once the predisposing factors were entered, gender was no longer a significant predictor.

**Table 3:** Hierarchical Regressions Predicting Engagement with MST-Related Medication Management Visits (*n*=1,021)

	Gender AOR (95% CI)	Predisposing Factors AOR (95% CI)	Need AOR (95% CI)	Enabling Factors AOR (95% CI)
Pseudo *R*^2^	.005	.010	.029	.031
Gender				
Men	ref	ref	ref	ref
Women	**0.69 (0.55–0.88)**	0.82 (0.65–1.05**)**	0.84 (0.66–1.08)	0.86 (0.67–1.10)
Mental health beliefs				
Low barrier class		ref	ref	ref
Stigma-related beliefs class		1.13 (0.83–1.53)	1.10 (0.81–1.49)	1.13 (0.82–1.55)
Negative perceptions of care class		0.99 (0.64–1.53)	0.95 (0.62–1.47)	1.00 (0.64–1.56)
High barrier class		0.99 (0.68–1.43)	0.93 (0.65–1.34)	0.99 (0.68–1.45)
Past-year mental health care use				
No		ref	ref	ref
Yes		**2.00 (1.55–2.58)**	**2.00 (1.54–2.59)**	**1.61 (1.05–2.46)**
Clinically significant symptoms				
No positive screen			ref	ref
Positive mental health screen			**1.62 (1.05–2.48)**	**2.01 (1.55–2.60)**
Logistical barriers to care				
None				ref
One logistical barrier				1.07 (0.80–1.44)
Two+ logistical barriers				0.80 (0.59–1.10)

*Note.* Adjusted for 128 stations. *AOR* adjusted odds ratio, *ref* reference category

Significant AORs are presented in bold

Table 4 displays results for individual psychotherapy. The *negative perceptions of care* class remained a significant predictor across all models, predicting a lower likelihood of engaging with individual psychotherapy. Endorsing two or more logistical barriers was associated with a lower likelihood of engagement.

**Table 4:** Hierarchical Regressions Predicting Engagement with MST-Related Individual Psychotherapy (*n*=1,021)

	Gender AOR (95% CI)	Predisposing Factors AOR (95% CI)	Need AOR (95% CI)	Enabling Factors AOR (95% CI)
Pseudo *R*^2^	.001	.013	.014	.022
Gender				
Men	ref	ref	ref	ref
Women	0.86 (0.67–1.11)	0.95 (0.73–1.23)	0.96 (0.74–1.25)	1.01 (0.78–1.30)
Mental health beliefs				
Low barrier class		ref	ref	ref
Stigma-related beliefs class		1.00 (0.71–1.41)	0.98 (0.70–1.38)	1.08 (0.76–1.53)
Negative perceptions of care class		**0.56 (0.38–0.84)**	**0.55 (0.37–0.82)**	**0.65 (0.43–0.96)**
High barrier class		0.74 (0.53–1.03)	0.72 (0.51–1.00)	0.85 (0.59–1.22)
Past-year mental health care use				
No		ref	ref	ref
Yes		**1.44 (1.09–1.92)**	**1.44 (1.09–1.91)**	1.37 (0.89–2.09)
Clinically significant symptoms				
No positive screen			ref	ref
Positive mental health screen			1.31 (0.85–2.01)	**1.41 (1.06–1.87)**
Logistical barriers to care				
None				ref
One logistical barrier				0.75 (0.54–1.03)
Two+ logistical barriers				**0.56 (0.38–0.83)**

*Note.* Adjusted for 128 stations. *AOR* adjusted odds ratio, *ref* reference category

Significant AORs are presented in bold

Lastly, Table 5 displays hierarchical regression models predicting engagement with group psychotherapy which produced similar findings to medication management visits. Women predicted a lower likelihood of engagement compared to men in the first model, which was no longer significant after predisposing factors were accounted for.

**Table 5:** Hierarchical Regressions Predicting Engagement with MST-Related Group Psychotherapy (*n*=1,021)

	Gender AOR (95% CI)	Predisposing Factors AOR (95% CI)	Need AOR (95% CI)	Enabling Factors AOR (95% CI)
Pseudo *R*^2^	.005	.081	.098	.118
Gender				
Men	ref	ref	ref	ref
Women	**0.61 (0.45–0.82)**	0.76 (0.55–1.03)	0.77 (0.59–1.09)	0.79 (0.58–1.08)
Mental health beliefs				
Low barrier class		ref	ref	ref
Stigma-related beliefs class		1.04 (0.75–1.44)	1.01 (0.73–1.40)	1.07 (0.75–1.51)
Negative perceptions of care class		0.60 (0.34–1.07)	0.59 (0.32–1.00)	0.64 (0.36–1.15)
High barrier class		1.08 (0.72–1.63)	1.03 (0.65–1.49)	1.15 (0.74–1.77)
Past-year mental health care use				
No		ref	ref	ref
Yes		**2.39 (1.79–3.20)**	**2.39 (1.11–2.89)**	**2.35 (1.75–3.17)**
Clinically significant symptoms				
No positive screen			ref	ref
Positive mental health screen			**1.58 (2.15–3.87)**	1.63 (0.97–2.75)
Logistical barriers to care				
None				ref
One logistical barrier				0.81 (0.59–1.12)
Two+ logistical barriers				0.70 (0.47–1.03)

*Note.* Adjusted for 128 stations. *AOR* adjusted odds ratio, *ref* reference category

Significant AORs are presented in bold

Notably, across all types of engagement and models, the *stigma-related beliefs* nor *high barrier* classes did not show any significant differences with engagement compared to the *low barrier* class.

## DISCUSSION

This study documents excellent access to specialized services for MST in the context of VHA’s comprehensive proactive policies for detection and treatment. Among Veterans who perceived a need for MST-related care, the vast majority (71.8%) accessed MST-related mental health care within 30 days, with the modal contacts representing same-day access. Nearly all treatment-seeking Veterans (88.8%) accessed care within the year following detection. These findings suggest very good access to sexual violence–related services. Given the intimate nature and the pervasive negative social reactions surrounding sexual assault, concerns about disclosure lead many survivors to delay or forgo care. These concerns appear to be well founded, as meta-analyses suggest that negative social reactions to assault disclosure serve to maintain or exacerbate mental health symptoms.^26^ Population-based data suggests that only 52% of sexual assault survivors ever receive assault-related mental health or health care services,^27^ though barriers such as insurance status and access to health care will also affect these estimates. Across users of a large, integrated civilian U.S. health care system, approximately half (54%) of women with documented sexual assault received mental health services following detection/documentation.^28^ Considering these prior estimates do not account for perceived need, the present findings underscore the excellent access to MST-related mental health care.

There are some potential explanations for the short median time to treatment access. Each VHA facility has mental health providers embedded in primary care and these providers often serve as a “warm handoff” to a mental health following positive mental health screens,^29^ resulting in improved access. Additionally, each facility has an MST coordinator, who is a provider with dedicated time for the MST program. In some settings, an initial referral to the MST coordinator may minimize typical mental health wait times for individuals with newly detected MST. Since these data were collected, the VHA has established a dashboard to track completed consults following MST detection. From our perspective, timely receipt of care following MST detection should be an expected outcome of VHA’s implementation of a comprehensive screening program, outreach, and provider education.

The social schema surrounding sexual assault appears to have a more pronounced effect on engagement with care as compared to access. In particular, negative perceptions of care (e.g., concerns about being blamed or not believed) appeared to be linked to a decreased likelihood of engagement with care. When all forms of MST-related care were considered together, the negative perceptions of care class emerged as a significant predictor of lower engagement, relative to the *low barrier* class, until logistical barriers were entered into the model. For individual psychotherapy, however, negative perceptions of care demonstrated consistent effects for a lower likelihood of engagement. It is possible that these effects are due to the importance of the therapeutic relationship, or the greater likelihood of discussing traumatic material in individual psychotherapy relative to medication management or group psychotherapy.

Prior research has demonstrated higher levels of negative mental health beliefs to be associated with more logistical barriers^30^ and symptoms of PTSD and depression.^31^ There may be a reciprocal relationship in that when Veterans experience logistical barriers and/or distress, this may maintain negative mental health–related beliefs, and negative mental health–related beliefs may increase the likelihood of experiencing logistical barriers and distress.^12^ Nonetheless, concerns such as provider reactions or confidentiality may be fruitful to address early on in psychotherapeutic treatment, to address any apprehensions that may be difficult to disclose, but that could negatively impact engagement. Interventions targeting mental health attitudes and barriers to treatment can increase retention in mental health services^14,32^ and may be especially impactful for women Veterans with PTSD.^33^ Specifically, interventions targeting negative perceptions of care may be helpful to implement during a first session to improve engagement and help all survivors get the mental health care that they seek and deserve.

This study used innovative methods to tailor treatment belief classes to known gender differences in treatment preferences, via multiple-group LCA.^12^ Main effects for gender, especially with respect to the experience of trauma, do not necessarily indicate essential differences between women and men, but can be a marker for interacting individual and contextual social factors.^34^ A gender variable can account for such effects due to omitted variable bias, when important determinants of outcomes that are correlated with gender are omitted.

Both women and men with a perceived need for MST-related care demonstrated good access to care. Women appeared to be less likely to engage in MST-related mental health care relative to men, though these gender differences appear to be accounted for by predisposing factors of use of mental health services in the year prior to MST detection and treatment beliefs. This is somewhat surprising as it is in contrast to the limited research documenting that men are significantly less likely than women to use health care services following newly detected MST.^35^ The difference in findings may be linked to our focus on MST-related care specifically and on a population with perceived need for care. Once perceived need is accounted for, men appear to be slightly more likely to engage in mental health care. The observed gender effects in our study appeared to be accounted for by prior mental health care use and treatment beliefs, which suggests that prior positive care experiences or beliefs may be more critical than gender in treatment engagement.

Although this study offers important information on access to care and which treatment barriers impact treatment engagement, there are some limitations to note. Our study surveyed Veterans with newly detected MST in 2013–2014. Since that time, the VHA has enhanced screening implementation to further promote access to care: the MST screen now formally prompts providers to query perceived need for care when MST is detected, and a clinician-facing dashboard is available to monitor timely receipt of care. Timely access to MST-related care is likely even higher than the rates documented in this study. The extent to which such policies also enhance engagement with care is worthy of further study, and our data suggest that communication tailored to treatment beliefs may be a promising avenue for interventions to promote engagement. Current symptoms of PTSD and depression were evaluated via screening questionnaires which are not confirming any diagnoses, although represent a good proxy for need. Using latent barrier classes to predict engagement holds the possibility of potential bias by uncertainty in latent class assignment; however, given good model fit with high entropy, this was likely negligible.

Though we prospectively ascertained MST-related care, we cannot rule out a bidirectional effect between treatment beliefs and treatment engagement. Because the large majority of Veterans who perceived a need for care received timely MST-related mental health care, these early treatment experiences, positive or negative, could have influenced the treatment beliefs reported on the survey. The majority of prior studies have looked at associations between mental health beliefs and assessed utilization retrospectively, not lending insight into directionality. One study that evaluated initiation of PTSD psychotherapy both retrospectively and prospectively to surveying beliefs (including negative perceptions of care and stigma) found no differences in belief impact across time periods.^17^ Additionally, sensitivity analyses suggest that the relationship we observed between treatment beliefs and engagement is not solely due to more positive beliefs among individuals with sufficient engagement to achieve clinical benefit.

Finally, this study is significant in that it is one of many that illustrates the pronounced impact that women’s health research can have for improving the health of both women *and* men. When the VHA was first mandated to detect and treat MST, programmatic oversight was housed in women’s health services, where there was a high concentration of expertise in sexual trauma. Over time, the VHA implemented screening and specialized treatment; it became clear that a substantial proportion of Veterans receiving care for MST were men. MST oversight was transitioned to mental health services, and implementation of education and clinical programs has increasingly recognized gender-tailored approaches as inherent to an effective approach to MST. While gender issues may be especially pronounced with respect to MST, MST is only one example of how the application of women’s health research can improve the effectiveness of care.
